# Identification of Neuroinflammation‐Related Diagnostic Biomarkers in Metabolic Dysfunction–Associated Steatohepatitis: An Integrated Bioinformatics and Machine Learning Approach

**DOI:** 10.1155/grp/2878164

**Published:** 2026-11-05

**Authors:** Yuliang Jia, Maocairang Li, Duo Ga, Xueyi Qian

**Affiliations:** ^1^ Department of Gastroenterology The First Affiliated Hospital of Wannan Medical University (Yijishan Hospital of Wannan Medical University) Wuhu Anhui China; ^2^ Department of Gastroenterology Shannan People′s Hospital Shannan Xizang Autonomous Region China

**Keywords:** candidate biomarkers, diagnostic model, machine learning, metabolic dysfunction–associated steatohepatitis, molecular docking, neuroinflammation-related gene programs

## Abstract

**Background:**

Metabolic dysfunction–associated steatohepatitis (MASH) is a liver disease that progresses and may be connected to neuroinflammation, though the specific molecular mechanisms are not yet understood. The objective of this research was to explore candidate diagnostic biomarkers related to neuroinflammation‐associated gene programs for MASH through integrated bioinformatics and machine learning, as a hypothesis‐generating study.

**Methods:**

Genes with differential expression (DEGs) were pinpointed from the GSE89632 dataset, which includes 39 MASH samples compared with 24 controls. Candidate genes were identified using weighted gene coexpression network analysis (WGCNA) and gene sets related to neuroinflammation. A diagnostic model was constructed using 81 machine learning algorithm combinations (filtered from an initial set of 113 by excluding models with fewer than 2 features) within the training set, and its performance was subsequently evaluated in GSE126848 (31 MASH vs. 26 controls) as an evaluation set. Because the evaluation set contributed to model selection, it does not represent a fully independent validation cohort. Regulatory network construction, drug prediction, and molecular docking were used for further analysis of key genes.

**Results:**

A total of 90 disease‐associated neuroinflammation‐related candidate genes were identified. Five candidate key genes (IRS1, HBEGF, MYC, CD274, and HMOX1) were screened, and a diagnostic model was constructed. The model achieved an AUC of 0.978 in the training set and 0.823 in the evaluation set; however, because the evaluation set contributed to model selection in the original analysis, the 0.823 estimate should be interpreted with caution. These findings are exploratory and require experimental validation.

**Conclusions:**

This exploratory study identified five neuroinflammation‐related candidate genes that may serve as candidate biomarkers for MASH. These findings provide candidate molecular links and potential therapeutic hypotheses, but require experimental validation and should be regarded as hypothesis‐generating rather than clinically actionable.

## 1. Background

Metabolic dysfunction–associated steatotic liver disease (MASLD) is a complex disorder marked by metabolic imbalance, liver fat buildup, oxidative stress, and persistent inflammation [1]. The range of the disease includes simple steatosis and extends to metabolic dysfunction–associated steatohepatitis (MASH), potentially leading to liver fibrosis, cirrhosis, and hepatocellular carcinoma [2–5]. With an estimated global prevalence of approximately 32.4%, MASLD has emerged as a leading cause of liver failure and indication for liver transplantation [6, 7].

Emerging evidence suggests that the pathological impact of MASH may extend beyond the liver to affect the central nervous system. Neuroinflammation, characterized by immune reactions involving activated glial cells and peripheral immune cells entering the central nervous system, is increasingly seen as a possible factor in neurological manifestations linked to MASH [8, 9]. Clinically, patients with cirrhosis frequently exhibit neuropsychiatric changes, including sleep disorders, anxiety, and depression, as part of hepatic encephalopathy [10, 11]. Histopathological evidence indicates that the cerebellum may be affected even before cirrhosis develops, with the MASH stage associated with microglial activation and increased release of proinflammatory cytokines [12]. A recent animal study demonstrated that mice fed a high‐fat diet combined with fructose developed anxiety‐ and depression‐like behaviors alongside low‐grade neuroinflammation [13]. Collectively, these observations suggest a potential bidirectional relationship between hepatic inflammation and neuroinflammatory processes. Nevertheless, the specific molecular mechanisms linking MASH to neuroinflammation remain largely unexplored, and reliable diagnostic biomarkers reflecting this association are currently lacking. Importantly, the present study does not directly measure neuroinflammation in the central nervous system; rather, it explores hepatic transcriptomic signatures related to neuroinflammation‐associated gene sets.

Recently, bioinformatics and machine learning techniques have become influential in discovering biomarkers and building diagnostic models [14]. These methods enable the integration of large‐scale transcriptomic data to identify key genes involved in disease pathogenesis [15, 16]. Furthermore, computational drug prediction combined with molecular docking offers efficient strategies for identifying potential therapeutic agents [17, 18].

In this study, we are aimed at (1) identifying differentially expressed genes in MASH that overlap with neuroinflammation‐related gene (NRG) sets through integrated bioinformatics analysis, (2) screening candidate key genes and construct a machine learning–based diagnostic model, (3) exploring the potential molecular mechanisms via regulatory network analyses, and (4) predicting candidate therapeutic drugs with computational docking predictions. Our research could shed new light on the potential molecular links between MASH and neuroinflammation‐related programs and suggest candidate biomarkers for further investigation.

## 2. Materials and Methods

### 2.1. Data Acquisition and Processing

Large‐scale transcriptomic datasets of liver tissue for MASLD were sourced from the Gene Expression Omnibus (GEO) database (https://www.ncbi.nlm.nih.gov/geo/). The GSE89632 training dataset was created with the GPL14951 platform (Illumina HumanHT‐12 WG‐DASL V4.0 R2 expression beadchip) and includes 39 MASH samples along with 24 normal controls [19, 20]. The evaluation set GSE126848 was generated using the GPL18573 platform (Illumina NextSeq 500), comprising 31 MASH samples and 26 normal controls [21]. Because GSE126848 contributed to model selection in the original analysis, it is referred to throughout this manuscript as an evaluation set rather than an independent validation cohort.

A total of 1336 NRGs were retrieved from the GeneCards database (https://www.genecards.org/) using a relevance score threshold of more than 1 for further analysis [22]. These genes represent database‐annotated neuroinflammation‐associated candidates rather than directly measured neuroinflammatory markers, and they were used to explore potential molecular links between MASH and neuroinflammation‐related programs.

#### 2.1.1. Data Preprocessing and Cross‐Platform Harmonization

Raw expression data for GSE89632 (GPL14951) and GSE126848 (GPL18573) were obtained from GEO. For each dataset, expression values were log_2_‐transformed after adding a small constant (1) to avoid undefined values for zero counts, and quantile‐normalized within each dataset using the normalizeBetweenArrays() function in the limma package (Version 3.60.4). Probes were mapped to gene symbols using the platform annotation files provided by GEO. When multiple probes mapped to the same gene symbol, the probe with the highest mean expression across samples was retained; all other probes were discarded. Genes not shared between the two datasets were removed prior to model training and evaluation.

To harmonize the two datasets across platforms, we first restricted the analysis to genes present in both datasets. Because the two datasets were generated on different platforms, we did not perform direct batch correction on the full expression matrix; instead, we *z*‐score–standardized each gene within each dataset separately (mean = 0, standard deviation = 1) before model training and evaluation. This within‐dataset standardization ensures that the model coefficients are not driven by platform‐specific scale differences. For the training set (GSE89632), the same standardization parameters (gene‐wise means and standard deviations) were applied to the evaluation set (GSE126848) to avoid information leakage from the evaluation set into the training process. No additional cross‐platform normalization (e.g., ComBat, and RUV) was applied, and this is acknowledged as a limitation.

#### 2.1.2. Availability of Clinical Metadata

We systematically reviewed the GEO records, the original publications, and their supplementary materials. Individual‐level demographic and clinicopathological data were not publicly accessible for either dataset. Specifically, age, sex, and BMI were not available at the individual level; histological diagnosis (MASH vs. normal control) was available as group labels and used in the analyses; NAS and fibrosis stage were not available at the individual level; metabolic parameters (HbA1c, lipid profile, fasting glucose, insulin, HOMA‐IR) were not available at the individual level; and medication use and alcohol consumption were not available for either dataset.

Although group‐level clinical data for a subset of GSE89632 participants have been reported in the original publication (Supplementary Table 1 of Pettinelli et al. 2022), these data could not be reliably linked to the individual GEO samples used in our analysis (39 MASH and 24 controls). Therefore, they could not be used for baseline comparison or adjustment.

Consequently, we were unable to construct a conventional cohort‐characteristics table or to adjust for fibrosis stage, metabolic status, medication use, or other potential confounders in our analyses. This is acknowledged as a limitation in Section 4.6.

### 2.2. Analysis of Differential Expression

The limma package (Version 3.60.4) in R (Version 4.4.1) was used to conduct differential expression analysis between MASH patients and normal controls in the GSE89632 training set [23]. Genes were considered differentially expressed if their adjusted *p* value was less than 0.05, and the absolute value of their log_2_ fold change was greater than 0.5. The ggplot2 package was used to visualize these DEGs [9, 24].

### 2.3. Analysis of Weighted Gene Coexpression Networks (WGCNA)

The WGCNA package (Version 1.73) was utilized to conduct WGCNA, aimed at identifying gene modules that are significantly linked to disease status. First, hierarchical clustering of samples was performed to identify and remove outlier samples. Second, the ideal soft‐thresholding power (*β*) was chosen according to the scale‐free fit index (*R* ^2^ > 0.85) and average connectivity. Third, an adjacency matrix was computed and converted into a topological overlap matrix (TOM). Fourth, modules were pinpointed using dynamic tree cutting with a minimum size of 50, and they were combined when their similarity was greater than 0.75. Ultimately, Pearson correlation analysis was employed to determine the relationships between module eigengenes and disease status [25]. Genes with module membership (MM) greater than 0.7 and gene significance (GS) exceeding 0.25 were chosen as hub genes for further analysis [15, 26].

### 2.4. Identification of Disease‐Associated NRGs

Genes related to neuroinflammation associated with diseases were identified by finding the common elements among NRGs, DEGs, and hub genes identified by WGCNA using the ggvenn package (Version 0.1.10).

### 2.5. Analysis of Gene Ontology (GO) and Kyoto Encyclopedia of Genes and Genomes (KEGG) Enrichment

The clusterProfiler package (Version 4.12.6) was used to conduct GO and KEGG enrichment analyses on genes related to neuroinflammation associated with the disease [27–29]. Terms with an adjusted *p* value below 0.05 were deemed significantly enriched. Visualization utilized the ggplot2 and enrichplot packages.

### 2.6. Protein–Protein Interaction (PPI) Network Analysis

A PPI network of disease‐associated NRGs was constructed using the STRING database (https://string-db.org/, Version 12.0) with a minimum interaction score threshold of 0.4. Cytoscape (Version 3.10.0) was used to visualize the network. Key hub genes were identified using five algorithms (MCC, degree, EPC, MNC, and betweenness) from the CytoHubba plugin, and the Top 30 genes from each were intersected to find key candidate genes [30].

### 2.7. Machine Learning for Key Gene Screening and Diagnostic Model Construction

Using 23 candidate hub genes, we applied an initial set of 113 combinations of machine learning algorithms derived from 12 base algorithms, including Lasso, Ridge, Elastic Net, Stepglm, SVM, glmBoost, LDA, plsRglm, random forest, GBM, XGBoost, and Naive Bayes [31]. Models with fewer than 2 features were automatically excluded, yielding the final 81 combinations used for model training. The models were trained using the GSE89632 dataset. In the original analysis, the optimal model was chosen based on the algorithm combination that achieved the highest average area under the curve (AUC) across both the training and evaluation datasets. This means that the GSE126848 dataset contributed to model selection and therefore does not represent a fully independent held‐out evaluation set; consequently, the reported evaluation AUC may be optimistic. In this revised manuscript, GSE126848 is described as an evaluation set, and its AUC should be interpreted with caution. The genes identified by this model were labeled as candidate key genes [32].

The final selected model was Stepglm[both] + plsRglm. The final model coefficients (on the *z*‐score–standardized gene expression scale) are as follows: Prediction formula (logit scale): logit(*p*(MASH)) = 0.938 + 0.670 × IRS1 − 0.890 × HBEGF − 1.152 × MYC − 0.536 × CD274 − 0.861 × HMOX1, where insulin receptor substrate 1 (IRS1), heparin‐binding EGF‐like growth factor (HBEGF), MYC, CD274, and heme oxygenase 1 (HMOX1) represent the *z*‐score–standardized expression values of the five candidate key genes. The predicted probability of MASH is given by *p*(MASH) = 1/(1 + exp(−logit(*p*(MASH)))). These coefficients are provided to facilitate reproducibility and external evaluation. They are derived from the training set and should not be interpreted as establishing causal or clinically actionable relationships.

A nomogram was constructed using the regplot package to visualize the prediction model. Model performance was evaluated using receiver operating characteristic (ROC) curves and AUC values calculated with the pROC package (Version 1.18.5). Confusion matrix analysis was performed to calculate accuracy, sensitivity, specificity, and F1 score. The rmda package was used to conduct decision curve analysis (DCA) for assessing clinical net benefit [25].

The permshap() function from the kernelshap package (Version 0.7.0) was used to conduct SHAP (SHapley Additive exPlanations) analysis for interpreting feature contributions. Visualization was performed using the shapviz package [33].

### 2.8. Expression Validation of Key Genes

The ggpubr package (Version 0.6.0) was used to conduct Wilcoxon rank‐sum tests to compare the expression levels of candidate key genes between MASH patients and normal controls in both the training and evaluation datasets [25].

### 2.9. Gene Set Enrichment Analysis (GSEA)

For each candidate key gene, Spearman correlation coefficients were calculated with all genes in the training set. The correlated gene lists were ranked by correlation coefficient, and KEGG pathway enrichment analysis was performed using the gseKEGG function from the clusterProfiler package. Significance was attributed to results with an adjusted *p* value below 0.05 and an absolute normalized enrichment score (NES) greater than 1 [34].

### 2.10. Regulatory Network Construction

#### 2.10.1. Transcription Factor‐Target Gene Network

The ChEA3 online tool (https://maayanlab.cloud/chea3/) was used to predict transcription factors for important genes [35]. The Top 10 TFs for each target gene were selected, and the TF‐target gene regulatory network was visualized using Cytoscape.

#### 2.10.2. ceRNA Regulatory Network

Candidate key gene‐targeting miRNAs were forecasted using DIANA‐microT (http://diana.imis.athena-innovation.gr/DianaTools/) and TargetScan (https://www.targetscan.org/). The intersection of predictions from both databases was used for subsequent analysis. Interactions between lncRNA and miRNA were sourced from the StarBase database (https://rnasysu.com/encori/), keeping entries with CLIP experiment support exceeding 15 and categorized as “lincRNA” [36]. A ternary regulatory network involving lncRNA, miRNA, and mRNA was built and displayed using a Sankey diagram created with the ggalluvial package (Version 0.12.5) [37, 38].

### 2.11. Drug Prediction and Molecular Docking

The DGIdb database (https://www.dgidb.org/, Version 5.0) was used to predict potential drugs targeting candidate key genes [17]. The 3D structures of proteins from candidate key genes were obtained from the Protein Data Bank (PDB) (https://www.rcsb.org/) [39]. Potential drug structures in both two and three dimensions were sourced from PubChem (https://pubchem.ncbi.nlm.nih.gov/) and DrugBank (https://go.drugbank.com/) [40]. CB‐Dock2 (https://cadd.labshare.cn/cb-dock2/) was used for molecular docking. Binding energies below −5.0 kcal/mol were used as a screening threshold to prioritize drug–target pairs for further investigation; this threshold does not by itself demonstrate true binding or therapeutic efficacy. No positive or negative docking controls, surface plasmon resonance (SPR), isothermal titration calorimetry (ITC), or functional assays were performed in this study [18, 41]. Docking results were visualized using PyMOL (Version 2.5).

### 2.12. Statistical Analysis

R software (Version 4.4.1) was used for all statistical analyses. The limma package, along with Benjamini–Hochberg correction for multiple comparisons, was employed for differential expression analysis. Wilcoxon rank‐sum tests were used to compare two groups, with a *p* value of less than 0.05 indicating statistical significance unless stated otherwise [42, 43]. Given the exploratory nature of the study and the modest sample size, all findings should be regarded as hypothesis‐generating.

## 3. Results

### 3.1. Identification of Differentially Expressed Genes

In the GSE89632 dataset, differential expression analysis between MASH patients and normal controls revealed 3011 DEGs, with 1395 genes being upregulated and 1616 genes being downregulated (Figure 1A,B).

**Figure 1:**
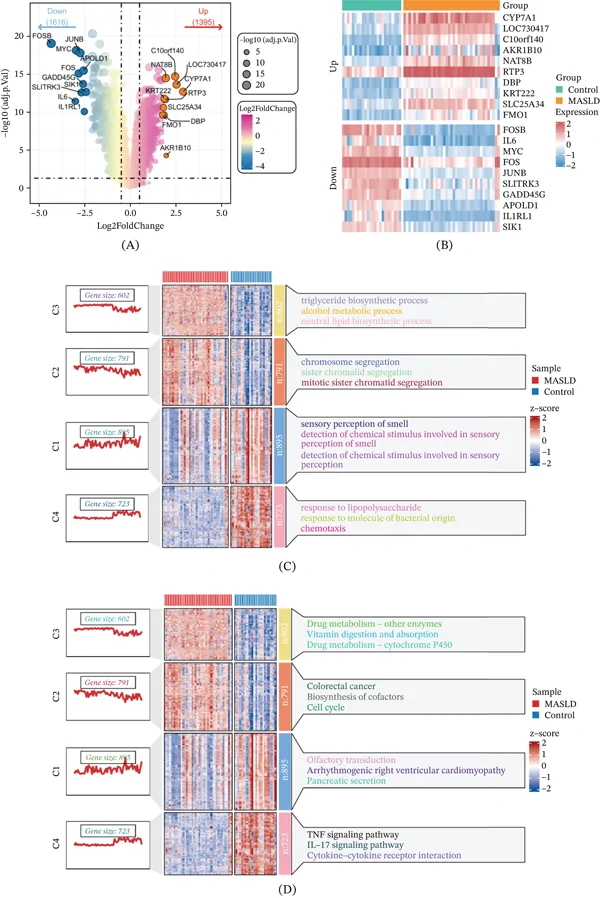
Identification of differentially expressed genes in MASH. (A) Volcano plot of DEGs (1395 upregulated in red, 1616 downregulated in blue). (B) Heatmap of Top 50 DEGs. (C) GOBP and (D) KEGG enrichment of four expression modules (C1–C4). (C) Combined heatmap and GOBP enrichment analysis showing the expression patterns (*z*‐score normalized) and Top 3 biological processes for each of the four gene modules (C1–C4). (D) Combined heatmap and KEGG enrichment analysis showing the expression patterns (*z*‐score normalized) and Top 3 pathways for each of the four gene modules (C1–C4)

Using mfuzz fuzzy clustering, the DEGs were divided into four expression modules (C1–C4). Modules C2 and C3 showed high expression in MASH patients, whereas C1 and C4 showed high expression in normal controls. KEGG analysis showed that module C4, which had high expression in normal controls, was significantly enriched in inflammatory pathways such as TNF signaling, IL‐17 signaling, and cytokine–cytokine receptor interaction (Figure 1C,D). This finding suggests that these inflammatory pathways may be dysregulated in MASH patients.

### 3.2. Identification of Disease‐Associated Module Genes by WGCNA

WGCNA was conducted to find gene modules linked to MASH disease status. Two outlier samples were excluded (Figure 2A), and the best soft‐thresholding power was identified as *β* = 6 (Figure 2B). Correlation analysis between module eigengenes and disease status showed that the blue module had the strongest positive correlation with MASH (correlation = 0.8, *p* = 2e − 10; Figure 2C). With thresholds set at MM > 0.7 and GS > 0.25, 1247 genes in the blue module were identified as disease‐associated hub genes (Figure 2D). The gene dendrograms before and after module merging, the clustering dendrogram of module eigengenes, and the heatmap of module eigengene adjacency are shown in Figure 2E–H.

**Figure 2:**
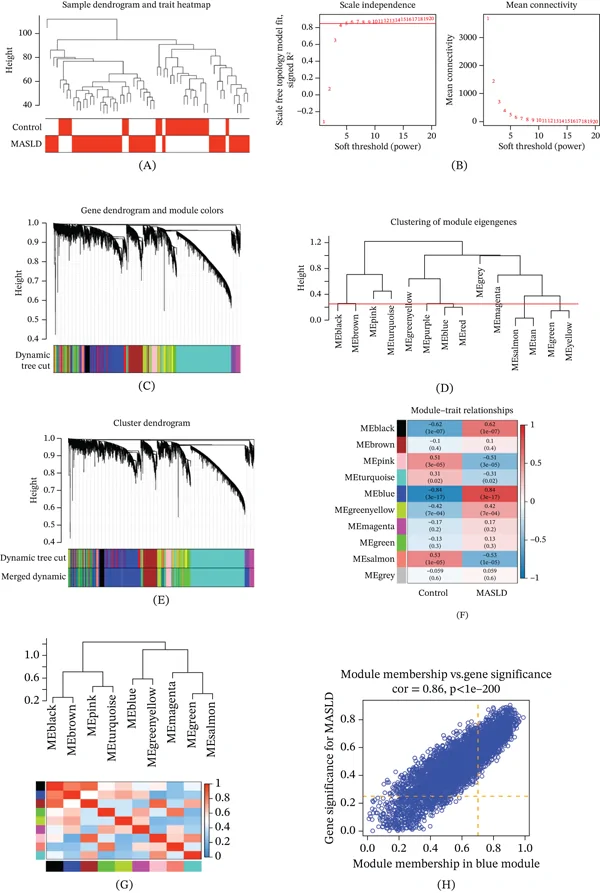
Weighted gene coexpression network analysis (WGCNA). (A) Sample clustering dendrogram (two outliers removed). (B) Selection of soft‐thresholding power (*β* = 6). (C) Heatmap of module–trait correlation indicating that the blue module had the highest positive correlation with MASH (correlation = 0.8, *p* = 2e − 10). (D) Scatter plot of module membership (MM) versus gene significance (GS) for the blue module (1247 genes selected). (E) Gene dendrogram with initial module colors (Dynamic Tree Cut). (F) Gene dendrogram with merged module colors. (G) Clustering dendrogram of module eigengenes (red line: cut height at 0.25). (H) Heatmap of module eigengene adjacency.

### 3.3. Identification of Disease‐Associated NRGs

Taking the intersection of the 1247 WGCNA‐identified module genes, 3,011 DEGs, and 1336 NRGs yielded 90 disease‐associated NRGs (Figure 3).

**Figure 3:**
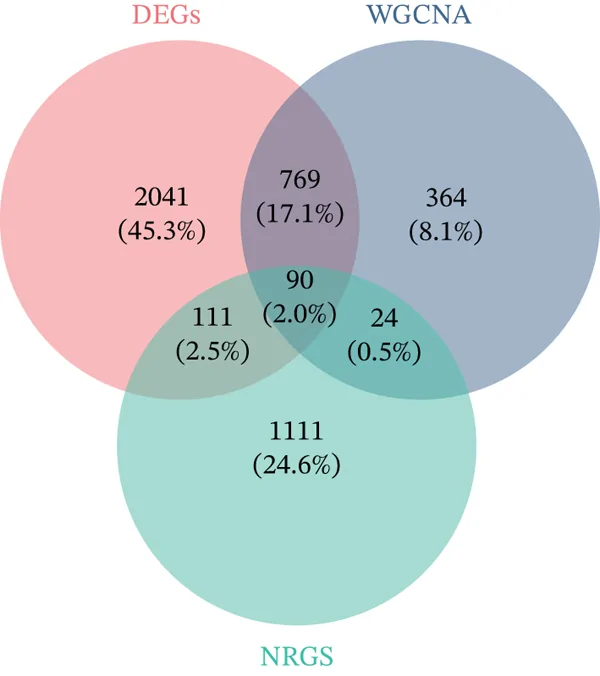
Identification of disease‐associated neuroinflammation‐related genes. A Venn diagram illustrates the overlap among DEGs (*n* = 3011), WGCNA module genes (*n* = 1247), and NRGs (*n* = 1336), identifying 90 shared genes.

### 3.4. GO and KEGG Enrichment Analysis of NRGs

We conducted GO and KEGG enrichment analyses to investigate the biological roles of the 90 genes linked to neuroinflammation and disease. GO analysis identified 1,033 significantly enriched terms, including 992 biological process (BP) terms, 15 cellular component (CC) terms, and 26 molecular function (MF) terms. In the BP category, the genes were significantly enriched in “response to lipopolysaccharide,” “regulation of inflammatory response,” and “cellular response to interleukin‐1.” In the CC category, significant enrichment was observed in “RNA polymerase II transcription regulator complex,” “membrane raft,” and “membrane microdomain.” Within the MF category, the genes were concentrated in “cytokine activity,” “G protein‐coupled receptor binding,” and “chemokine activity” (Figure 4A).

**Figure 4:**
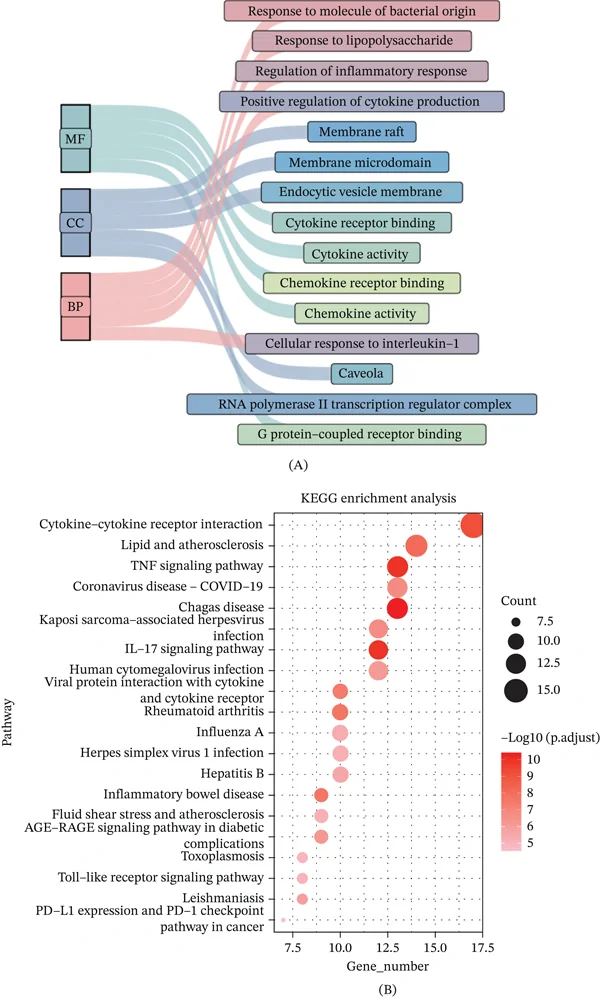
Functional enrichment analysis. (A) GO enrichment analysis showing significantly enriched terms categorized by biological process (BP), cellular component (CC), and molecular function (MF). Dot size represents gene count, and color represents adjusted *p* value. (B) The KEGG pathway enrichment analysis highlights the Top 20 pathways with significant enrichment. The *x*‐axis indicates the number of genes, the *y*‐axis lists the pathway names, and the color of the dots shows the adjusted *p* value.

KEGG enrichment analysis identified 83 significantly enriched pathways. The Top 20 pathways are shown in Figure 4B. The genes were enriched in the “cytokine‐cytokine receptor interaction,” “TNF signaling pathway,” “IL‐17 signaling pathway,” and “Toll‐like receptor signaling pathway” (Figure 4B). These are shared inflammatory pathways that may also be relevant to neuroinflammation, but they are not neuroinflammation‐specific.

### 3.5. PPI Network and Hub Gene Identification

A PPI network was built with 90 genes related to neuroinflammation and disease, consisting of 77 nodes and 510 edges after isolated nodes were removed (Figure 5A). To identify hub genes within this network, we used five algorithms (MCC, Degree, EPC, MNC, and betweenness) from the CytoHubba plugin. Intersecting the Top 30 genes from each algorithm resulted in 23 shared candidate hub genes (Figure 5B). These candidate hub genes should be interpreted as network‐based candidates rather than validated functional hubs.

**Figure 5:**
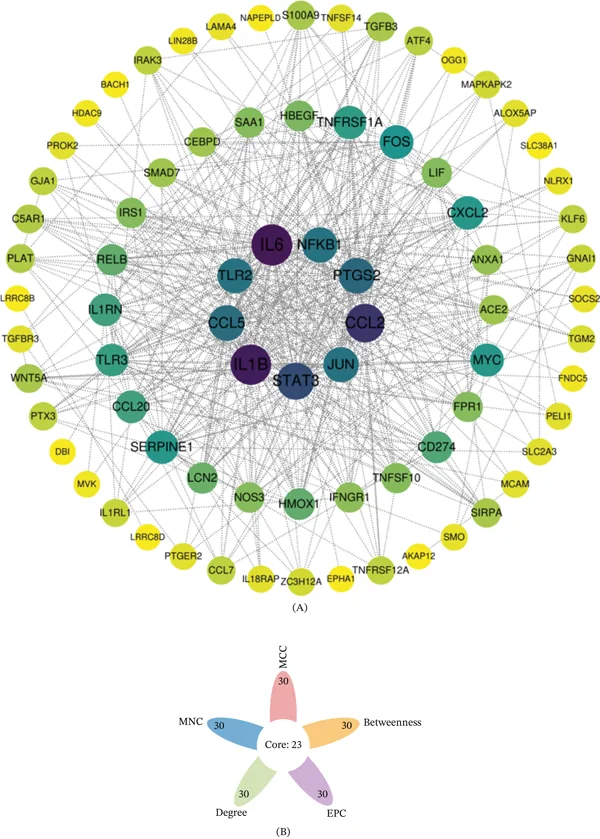
Identification of hub genes and PPI network. (A) PPI network of 77 nodes and 510 edges constructed using the STRING database. Nodes represent genes, and edges represent protein–protein interactions. Node size and color intensity are proportional to the degree of connectivity. (B) Intersection plot (flower plot) showing the overlap of Top 30 genes identified by five CytoHubba algorithms (MCC, Degree, EPC, MNC, and Betweenness), yielding 23 common candidate hub genes.

### 3.6. Key Gene Screening and Diagnostic Model Construction

Using the 23 candidate hub genes, we applied an initial set of 113 machine learning algorithm combinations; after excluding models with fewer than 2 features, 81 combinations remained. The Stepglm[both] + plsRglm combination achieved the highest average AUC (0.9) across the training and evaluation sets (Figure 6A), and identified five candidate key genes: IRS1, HBEGF, MYC, CD274, and HMOX1. Because the evaluation set contributed to model selection, this estimate may be optimistic.

Figure 6Machine learning analysis and diagnostic model. (A) Performance heatmap of 81 algorithm combinations. The optimal combination was Stepglm[both] + plsRglm, with a mean AUC of 0.9 across the training and evaluation sets; because the evaluation set (GSE126848) contributed to model selection, this estimate may be optimistic. (B) ROC curve of the diagnostic model in the training set (AUC = 0.978, 95% CI: 0.944–1.000). (C) ROC curve of the diagnostic model in the evaluation set (AUC = 0.823, 95% CI: 0.713–0.932). (D) ROC curves for each of the five candidate key genes in the training set (IRS1: 0.889, HBEGF: 0.955, MYC: 0.988, CD274: 0.937, and HMOX1: 0.934). (E) ROC curves for each of the five candidate key genes in the evaluation set (IRS1: 0.925, HBEGF: 0.611, MYC: 0.702, CD274: 0.864, and HMOX1: 0.935). (F) Confusion matrix of the diagnostic model in the training set (accuracy = 0.94; sensitivity = 0.95; specificity = 0.92; F1 score = 0.95). (G) Confusion matrix of the diagnostic model in the evaluation set (accuracy = 0.77; sensitivity = 0.74; specificity = 0.84; F1 score = 0.81). (H) Nomogram for predicting MASH probability based on the five candidate key genes; a representative sample is highlighted in red. (I) Decision curve analysis showing the net benefit of the nomogram compared with “treat all” and “treat none” strategies across different threshold probabilities. (J) SHAP bar plot showing the global importance of each feature, ranked by mean absolute SHAP value (MYC: 1.062, HBEGF: 0.795, HMOX1: 0.706, IRS1: 0.520, and CD274: 0.437). (K) SHAP bee swarm plot showing the distribution of SHAP values for each feature across samples; color represents feature value (high vs. low expression). These model‐based directions do not establish biological risk or protective effects.

**Figure:**
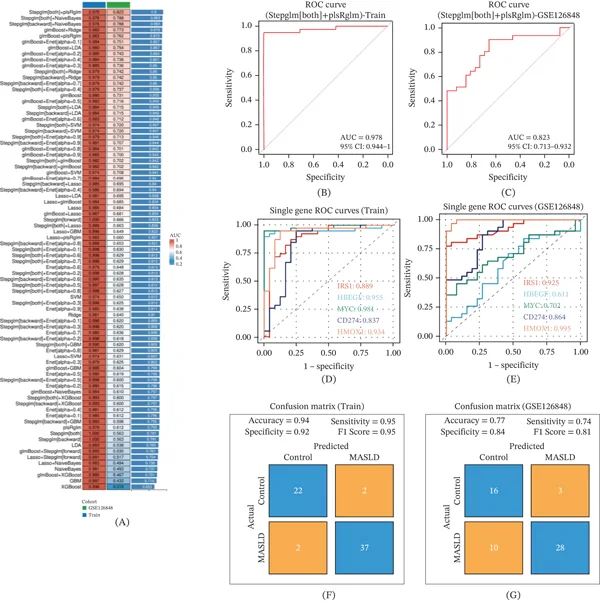

**Figure:**
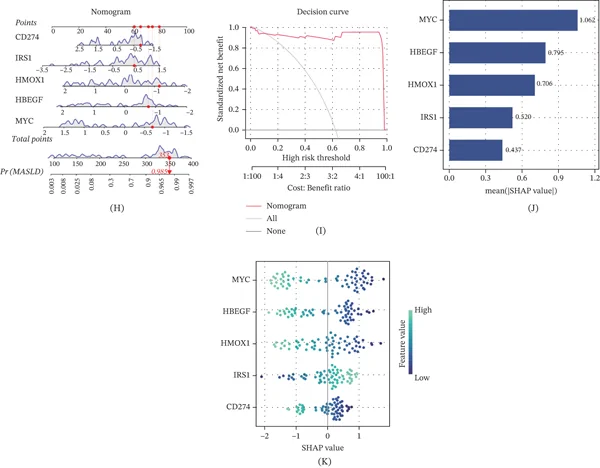

The diagnostic model achieved AUCs of 0.978 (95% CI: 0.944–1.000) in the training set (Figure 6B) and 0.823 (95% CI: 0.713–0.932) in the evaluation set (Figure 6C). Single‐gene AUCs in the training set were 0.889 (IRS1), 0.955 (HBEGF), 0.988 (MYC), 0.937 (CD274), and 0.934 (HMOX1) (Figure 6D); in the evaluation set, they were 0.925, 0.611, 0.702, 0.864, and 0.935, respectively (Figure 6E). Confusion matrix analysis showed accuracy 0.94, sensitivity 0.95, specificity 0.92, and F1 0.95 in the training set (Figure 6F), and accuracy 0.77, sensitivity 0.74, specificity 0.84, and F1 0.81 in the evaluation set (Figure 6G).

A nomogram was constructed to visualize the predicted probability of MASH (Figure 6H), and DCA showed a greater net benefit than “treat all” or “treat none” strategies (Figure 6I). SHAP analysis ranked MYC (1.062), HBEGF (0.795), and HMOX1 (0.706) as the largest contributors, followed by IRS1 (0.520) and CD274 (0.437) (Figure 6J). The SHAP bee swarm plot showed that high IRS1 expression corresponded to positive SHAP values, whereas high expression of MYC, HBEGF, HMOX1, and CD274 corresponded to negative SHAP values (Figure 6K); these model‐based directions do not establish biological risk or protective effects.

### 3.7. Expression Validation of Candidate Key Genes

To validate the expression patterns of the five candidate key genes, we compared their expression levels between MASH patients and normal controls in both the training set (GSE89632) and the evaluation set (GSE126848) using Wilcoxon rank‐sum tests (Figure 7). In the training set (Figure 7A), IRS1 was significantly upregulated in MASH patients compared with normal controls, whereas HBEGF, MYC, CD274, and HMOX1 were significantly downregulated (all *p* < 0.0001). In the evaluation set (Figure 7B), the same direction of change was observed for all five genes: IRS1 was significantly upregulated in MASH patients, whereas HBEGF, MYC, CD274, and HMOX1 were significantly downregulated (IRS1: *p* < 0.0001; HBEGF: *p* < 0.0001; MYC: *p* = 0.0021; CD274: *p* < 0.0001; HMOX1: *p* < 0.0001). These results indicate that the expression patterns of the five candidate key genes are consistent between the training and evaluation datasets, supporting their potential utility as candidate biomarkers for MASH. However, because both datasets are cross‐sectional and the evaluation set contributed to model selection, these findings should be regarded as exploratory and require validation in independent cohorts.

**Figure 7:**
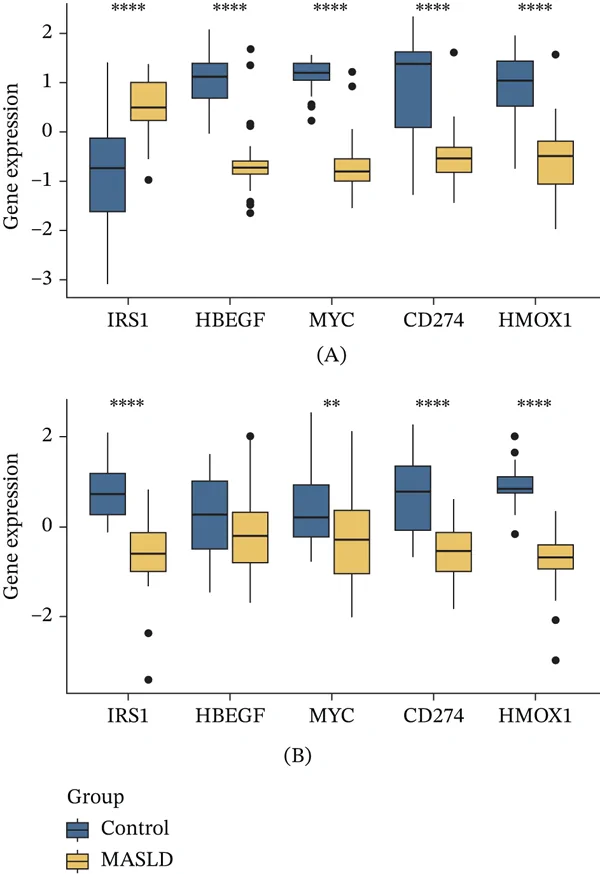
Expression validation of the five candidate key genes. (A) Box plots showing the expression levels of IRS1, HBEGF, MYC, CD274, and HMOX1 in the training set (GSE89632). (B) Box plots showing the expression levels of the same five genes in the evaluation set (GSE126848). Blue boxes represent normal controls; yellow boxes represent MASH patients. The horizontal line within each box represents the median, and the whiskers represent the interquartile range. Statistical significance was assessed using Wilcoxon rank‐sum tests ( ^∗∗∗∗^ *p* < 0.0001;  ^∗∗^ *p* < 0.01).

### 3.8. GSEA

We conducted GSEA to investigate the biological pathways linked to each candidate key gene. Taking MYC as an example (Figure 8), MYC was positively correlated with inflammatory pathways including the TNF signaling pathway (NES = 2.15, adjusted *p* = 0.001), IL‐17 signaling pathway (NES = 1.98, adjusted *p* = 0.003), and NF‐*κ*B signaling pathway (NES = 1.87, adjusted *p* = 0.008). In contrast, MYC was negatively correlated with metabolic pathways including valine, leucine, and isoleucine degradation (NES = −2.01, adjusted *p* = 0.002) and oxidative phosphorylation (NES = −1.79, adjusted *p* = 0.012). These results suggest that MYC expression is associated with inflammatory pathways and inversely associated with metabolic pathways in MASH. Whether MYC causally promotes inflammation or suppresses metabolic processes cannot be determined from this correlational analysis. The GSEA results for the remaining four candidate key genes (IRS1, HBEGF, CD274, and HMOX1) are presented in Figure S3.

**Figure 8:**
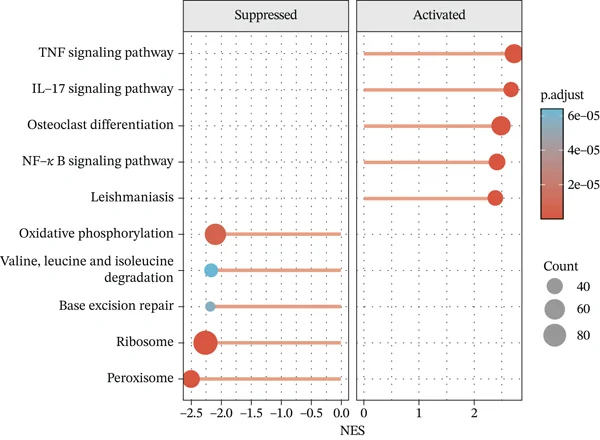
Gene Set Enrichment Analysis (GSEA) for MYC. MYC positively correlated with inflammatory pathways (TNF, IL‐17, and NF‐*κ*B) and negatively correlated with metabolic pathways (valine/leucine/isoleucine degradation and oxidative phosphorylation).

### 3.9. Regulatory Network of Transcription Factors and Target Genes

Using the ChEA3 online tool, we predicted the Top 10 transcription factors for each candidate key gene. The resulting TF‐target gene regulatory network (Figure 9) showed that several transcription factors may simultaneously regulate multiple candidate key genes. For instance, NR4A3 was anticipated to control MYC, CD274, HBEGF, and HMOX1, whereas HIVEP1 was expected to regulate MYC, CD274, HBEGF, and IRS1.

**Figure 9:**
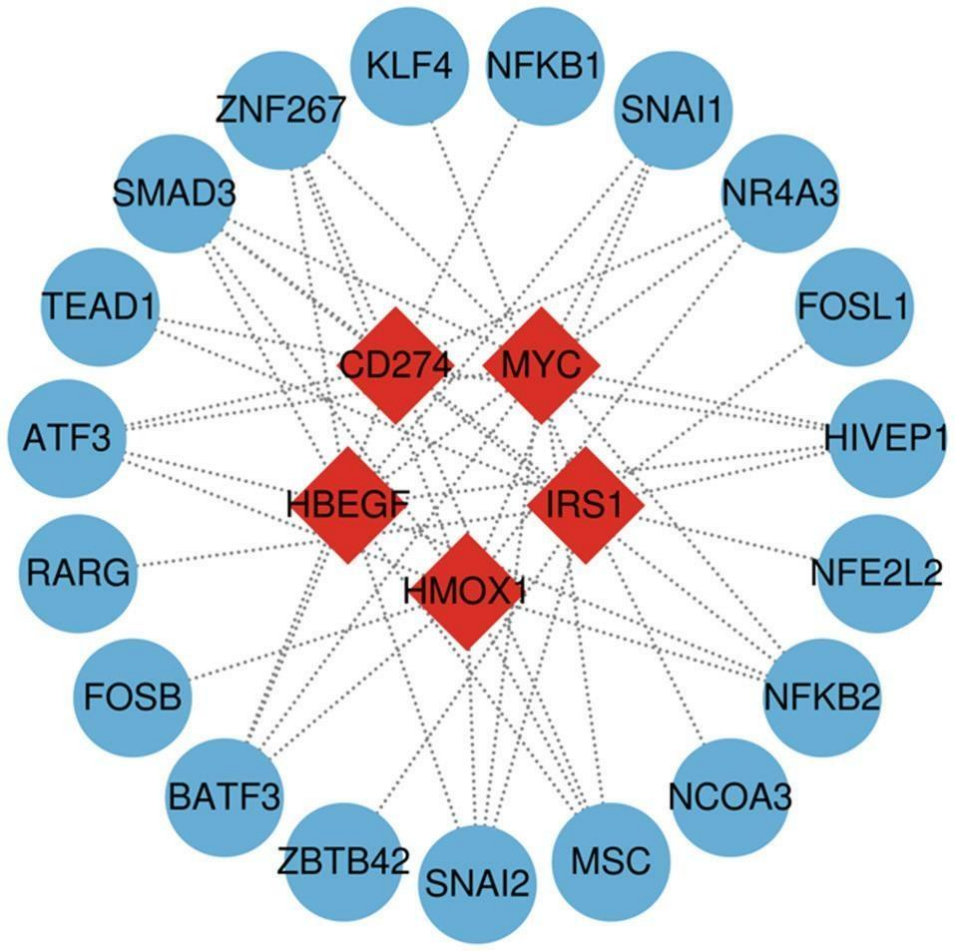
Transcription factor‐target gene regulatory network. Red diamonds: candidate key genes (IRS1, HBEGF, MYC, CD274, and HMOX1). Blue circles: transcription factors (Top 10 per target). Edges indicate regulatory relationships. Transcription factors regulating multiple target genes include NR4A3 (regulates MYC, CD274, HBEGF, and HMOX1) and HIVEP1 (regulates MYC, CD274, HBEGF, and IRS1).

### 3.10. ceRNA Regulatory Network

miRNA prediction using DIANA‐microT and TargetScan identified 29 common miRNAs (Figure S1A). We developed a lncRNA‐miRNA‐mRNA regulatory network using lncRNA‐miRNA interactions from StarBase. The Sankey diagram (Figure S1B) showed that 7 lncRNAs targeted 22 miRNAs, which in turn regulated three of the five candidate key genes: CD274, HBEGF, and IRS1. No such regulatory relationships were predicted for MYC or HMOX1, suggesting that these two genes may be primarily regulated at the transcriptional level.

### 3.11. Drug Prediction and Molecular Docking

Using the DGIdb database, we predicted 60 approved drugs as potential therapeutic agents targeting the five candidate key genes, including 7 targeting IRS1, 9 targeting HBEGF, 29 targeting MYC, 9 targeting CD274, and 6 targeting HMOX1 (Table S1). A drug–gene network was constructed to visualize these interactions (Figure S2).

Molecular docking was performed for the top‐scoring drug‐target pairs. The binding energies are shown in Table 1. IRS1 showed predicted binding affinity with Glimepiride (−6.8 kcal/mol), HBEGF with Alectinib (−6.9 kcal/mol), and MYC with Quinapril (−6.2 kcal/mol). These docking results are computational predictions only and were not validated by SPR, ITC, or functional assays.

**Table 1:** Predicted binding energies of candidate genes with potential drugs.

Gene	Drug	Binding energy (kcal/mol)
IRS1	Glimepiride	−6.8
HBEGF	Alectinib	−6.9
MYC	Quinapril	−6.2

*Note:* Only drug‐target pairs with a predicted binding energy below −5.0 kcal/mol are listed. These values are computational predictions and do not demonstrate true binding or therapeutic efficacy.

Visualization of docking results (Figure 10) showed predicted interaction: IRS1 with Glimepiride formed hydrogen bonds at residue MET‐260, HBEGF with Alectinib at residues LYS‐111 and TYR‐112, and MYC with Quinapril at residue TYR‐55.

**Figure 10:**
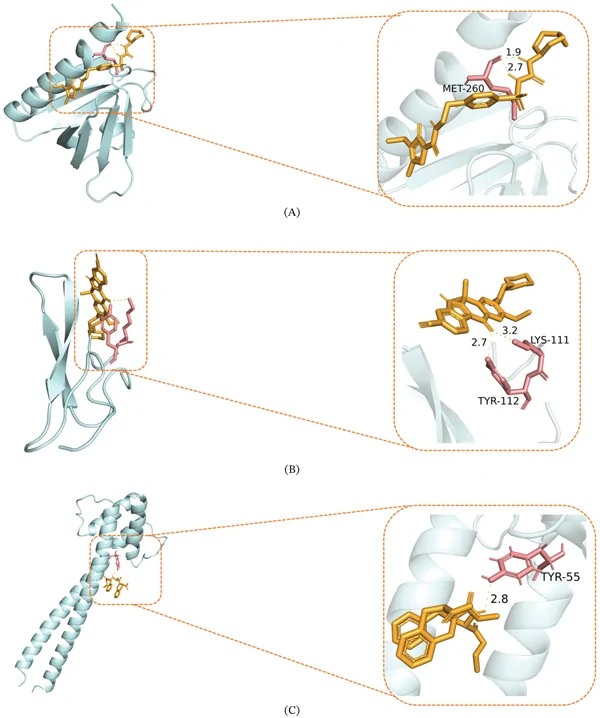
Computational molecular docking predictions. (A) IRS1‐Glimepiride (binding energy: −6.8 kcal/mol; hydrogen bond at MET‐260). (B) HBEGF‐Alectinib (−6.9 kcal/mol; LYS‐111, TYR‐112). (C) MYC‐Quinapril (−6.2 kcal/mol; TYR‐55). Hydrogen bonds are represented by yellow dashed lines. These are predicted interactions without experimental validation.

## 4. Discussion

In this exploratory research, we combined bioinformatics with machine learning techniques to explore candidate diagnostic biomarkers related to neuroinflammation for MASH. Five candidate genes (IRS1, HBEGF, MYC, CD274, and HMOX1) were identified, and a diagnostic model was developed, showing exploratory predictive performance (AUC = 0.978 in the training set and 0.823 in the evaluation set). We further explored the potential molecular mechanisms underlying these genes through GSEA and regulatory network analyses, predicted potential therapeutic drugs, and generated computational docking predictions for drug‐target interactions. These results provide candidate molecular links between MASH and NRG programs, which require direct CNS validation.

### 4.1. Summary of Key Findings

Our exploratory study yielded four main findings. First, we identified 90 genes that overlap between MASH‐associated and NRG sets and that were enriched in shared inflammatory pathways, suggesting a possible molecular connection between MASH and neuroinflammation‐related programs. Second, we screened five candidate key genes and constructed a diagnostic model whose performance was evaluated in a separate evaluation set. Third, within the model, IRS1 showed a positive SHAP contribution, whereas HBEGF, MYC, CD274, and HMOX1 showed negative SHAP contributions; these model‐based directions do not establish biological risk or protective effects. Fourth, we predicted 60 approved drugs as potential repurposing candidates and generated computational docking predictions for three drug–target pairs, which require experimental validation.

### 4.2. Biological Significance of the Five Candidate Key Genes

IRS1 is a critical mediator of insulin signaling. The observed upregulation of IRS1 in MASH patients aligns with the insulin resistance characteristic of MASLD. Although IRS1 is typically associated with impaired insulin signaling, its increased expression in MASH may represent a compensatory response to chronic hyperinsulinemia. Our SHAP analysis showed that IRS1 had a positive contribution within the model, which is consistent with its established role in metabolic dysfunction. Previous studies have shown that IRS1 dysfunction is associated with hepatic steatosis and inflammation [1, 44]. Whether IRS1 also links hepatic pathology to neuroinflammatory processes remains a hypothesis that requires experimental validation.

HBEGF is involved in tissue repair, cell proliferation, and inflammation. The downregulation of HBEGF in MASH patients observed in our study is consistent with a recent report showing reduced HBEGF expression in fibrotic liver tissues [45]. HBEGF promotes hepatocyte proliferation and liver regeneration following injury [46]. Therefore, its downregulation in MASH may be associated with impaired hepatic regenerative capacity and potentially with disease progression, although this hypothesis requires functional validation.

MYC is a proto‐oncogene that regulates cell proliferation, metabolism, and apoptosis. The downregulation of MYC in MASH patients was unexpected given its well‐established role in promoting cell proliferation. However, GSEA revealed that MYC was positively correlated with inflammatory pathways (TNF, IL‐17, and NF‐*κ*B) and negatively correlated with metabolic pathways. These results suggest that MYC expression is associated with inflammatory pathways and inversely associated with metabolic pathways in MASH. Whether MYC causally promotes inflammation or suppresses metabolic processes cannot be determined from this correlational analysis.

CD274 (PD‐L1) is an immune checkpoint molecule that negatively regulates T cell activation. The downregulation of CD274 in MASH patients may contribute to chronic inflammation by reducing immune suppression. In line with this, earlier research has indicated that the disruption of the PD‐1/PD‐L1 pathway plays a role in the advancement of chronic liver diseases [47]. Our findings raise the hypothesis that restoring CD274 expression might be beneficial in MASH, but this possibility requires experimental testing.

HMOX1 is a cytoprotective enzyme with anti‐inflammatory, antioxidant, and anti‐apoptotic properties. The downregulation of HMOX1 in MASH patients may compromise antioxidant defenses, potentially exacerbating oxidative stress and inflammation. Numerous studies have demonstrated that HMOX1 induction protects against liver injury in various models. Our findings are consistent with the possibility that HMOX1 downregulation may be involved in MASH pathogenesis and suggest that HMOX1 activators could be explored as candidate therapeutic agents in future studies.

### 4.3. Neuroinflammation‐Related Programs: A Potential Link in MASH Pathogenesis

The enrichment of our identified genes in inflammatory pathways, including TNF, IL‐17, and toll‐like receptor signaling, provides molecular evidence that MASH‐associated hepatic transcriptomic changes overlap with NRG programs. However, these pathways are shared inflammatory pathways rather than neuroinflammation‐specific signatures, and no brain or CNS tissue was analyzed in this study. Previous clinical studies have documented neuropsychiatric manifestations in patients with liver disease, including sleep disorders, anxiety, and depression [11, 48]. Histopathological examination of postmortem cerebellar tissues from patients with steatohepatitis revealed microglial activation and increased proinflammatory cytokine release [12]. Animal studies have further demonstrated that diet‐induced MASLD is associated with anxiety‐ and depression‐like behaviors and low‐grade neuroinflammation [13]. Our results are consistent with these observations but do not establish a direct mechanistic link.

The precise mechanisms by which hepatic inflammation may lead to neuroinflammation remain incompletely understood. Several hypotheses have been proposed: (1) systemic inflammation and circulating proinflammatory cytokines might penetrate the blood–brain barrier and stimulate microglia [8], (2) gut dysbiosis associated with MASH may produce neuroactive metabolites that affect brain function [49], and (3) hepatic encephalopathy–related metabolites such as ammonia may directly impair neuronal function [10]. Future studies are needed to test whether the five candidate genes identified here are involved in these pathways.

### 4.4. Comparison With Previous Studies

Several studies have previously attempted to identify diagnostic biomarkers for MASH using bioinformatics approaches. For example, Xia et al. [50] constructed a diagnostic model for nonalcoholic steatohepatitis using machine learning. However, to our knowledge, none of these studies specifically focused on NRGs. To our knowledge, this is among the first studies to explore the transcriptomic overlap between MASH and NRG sets. However, this is an exploratory bioinformatics study and does not provide direct evidence of neuroinflammation in MASH patients. Compared with previous bioinformatics studies, our approach has several strengths. First, we included a separate evaluation set to assess our findings; however, because this evaluation set contributed to model selection, the reported evaluation performance should be regarded as exploratory and requires confirmation in truly independent cohorts. Second, we applied 81 machine learning algorithm combinations (filtered from an initial 113) to explore robust gene selection. Third, we performed molecular docking to generate computational predictions for potential drug‐target interactions, providing translational relevance.

### 4.5. Clinical Implications

The diagnostic model, which relies on five candidate key genes, reached AUC values of 0.978 in the training set and 0.823 in the evaluation set, demonstrating exploratory discriminatory capability. The nomogram we developed serves as a research tool for predicting MASH probability using the expression levels of these five genes. However, because the evaluation set contributed to model selection and no experimental validation was performed, this model should not be presented as suitable for early clinical screening. If confirmed in future studies with larger and truly independent cohorts and experimental validation, this model might eventually help identify patients at high risk for MASH. At present, it should be regarded as a hypothesis‐generating research tool rather than a clinical diagnostic test.

Furthermore, the predicted drugs—particularly Glimepiride (targeting IRS1), Alectinib (targeting HBEGF), and Quinapril (targeting MYC)—represent potential repurposing candidates requiring preclinical testing. These predictions are based on computational drug–gene interaction and docking analyses and have not been validated experimentally. Glimeperide is an FDA‐approved sulfonylurea for Type 2 diabetes, and given the association between MASH and Type 2 diabetes [2], it could be explored in future preclinical studies. Alectinib, an ALK inhibitor approved for nonsmall cell lung cancer, has an unclear relationship with MASH and requires further investigation. Quniapril is an ACE inhibitor used for hypertension; ACE inhibitors have been reported to have anti‐inflammatory effects [17], but whether they are beneficial in MASH remains unknown.

### 4.6. Limitations

Several important limitations should be acknowledged. First, this study is purely computational and lacks experimental validation. No qPCR, Western blot, immunohistochemistry, cell‐based, or animal experiments were performed. All findings, including the five candidate genes and the diagnostic model, are hypothesis‐generating and require experimental confirmation. Second, this study used liver transcriptomic data and did not analyze brain tissue, cerebrospinal fluid, or neuroimaging data. The NRG set was derived from database annotations, and the enriched pathways are shared inflammatory pathways rather than neuroinflammation‐specific signatures. Therefore, no direct conclusion about neuroinflammation in MASH patients can be drawn. Third, the sample sizes were modest (39 MASH and 24 controls in the training set; 31 MASH and 26 controls in the evaluation set), resulting in a high complexity‐to‐sample‐size ratio for WGCNA, PPI hub selection, and 81 machine learning combinations (filtered from an initial 113). This increases the risk of overfitting. Fourth, the evaluation set was used in the original model selection process, which may have inflated the reported evaluation AUC; the 0.823 estimate should therefore be interpreted with caution and confirmed in truly independent cohorts. Fifth, the molecular docking results are computational predictions without positive or negative controls, SPR, ITC, or functional validation. The −5.0 kcal/mol threshold is only a screening cutoff and does not demonstrate true binding or therapeutic efficacy. Sixth, the causal relationships between the five candidate genes and MASH pathogenesis cannot be determined from correlational analyses. Seventh, individual‐level clinical metadata were not publicly available for either dataset. We systematically searched the GEO records, the original publications, and their supplementary materials, but confirmed that variables such as age, sex, BMI, NAS, fibrosis stage, metabolic parameters, medication use, and alcohol consumption were not accessible at the individual level. Only group labels (MASH vs. normal control) were available. We were therefore unable to construct a conventional cohort‐characteristics table or to adjust for fibrosis stage, metabolic status, medication use, or other potential confounders. The inability to adjust for these confounders is acknowledged as a limitation.

### 4.7. Future Directions

Several directions for future research emerge from this exploratory study. First, prospective cohort studies with larger sample sizes and detailed clinical metadata are needed to validate the diagnostic model in truly independent populations. Second, experimental studies using cell culture (e.g., hepatocytes and microglia) and animal models (e.g., high‐fat diet‐fed mice) should be conducted to elucidate the functional roles of IRS1, HBEGF, MYC, CD274, and HMOX1 in MASH pathogenesis. Third, the potential repurposing of Glimepiride, Alectinib, and Quniapril for MASH should be tested in preclinical models. Fourth, direct examination of neurological tissues or cerebrospinal fluid from MASH patients would help establish the clinical relevance of the NRGs identified in this study. Fifth, single‐cell transcriptomic studies could reveal cell type–specific expression patterns of these candidate genes in both liver and brain tissues.

## 5. Conclusions

This exploratory study identified five neuroinflammation‐related candidate genes (IRS1, HBEGF, MYC, CD274, and HMOX1) that may serve as candidate biomarkers for MASH. A machine learning–based diagnostic model including these five genes showed promising predictive performance in the training set and was evaluated in a separate evaluation set, although the evaluation AUC may be optimistic because the evaluation set contributed to model selection in the original analysis. Within the model, IRS1 showed a positive SHAP contribution, whereas HBEGF, MYC, CD274, and HMOX1 showed negative SHAP contributions; these model‐based directions do not establish biological risk or protective effects. GSEA and regulatory network analyses provided hypotheses about how these genes might be connected to MASH and neuroinflammation‐related programs at the molecular level. Finally, we predicted 60 approved drugs as potential repurposing candidates and generated computational docking predictions for three drug–target pairs, which require experimental validation. These results lay the groundwork for future experimental research and should be regarded as hypothesis‐generating rather than clinically actionable.

## Author Contributions

Xueyi Qian: conceptualization and funding acquisition; Maocairang Li: data analysis; Duo Ga: validation; Yuliang Jia: writing—original draft and funding acquisition; all authors: writing—review and editing.

## Funding

This study was supported by the Natural Science Foundation of Xizang Autonomous Region (XZ2024ZR‐ZY075(Z)) and Yuliang Jia and the Key Project of Wannan Medical (WK2024ZZD13).

## Disclosure

The authors take full responsibility for the integrity and final content of the published article.

## Ethics Statement

This study was conducted using publicly available datasets from the Gene Expression Omnibus (GEO) database, specifically GSE89632 and GSE126848. All original studies included in these datasets had obtained appropriate ethical approval and informed consent from their respective institutional review boards (IRBs) or ethics committees. As this study involved only secondary analysis of deidentified, publicly accessible data, it did not constitute human subjects research requiring additional ethical approval. According to the regulations of the Ethics Committee of The First Affiliated Hospital of Wannan Medical University, studies based on publicly available anonymized data are exempt from further ethics review (exemption number: “not applicable”]). All analyses were performed in accordance with relevant guidelines and regulations. The requirement for informed consent was waived by the ethics committee because the data were anonymized and publicly available.

## Consent

The authors have nothing to report.

## Conflicts of Interest

The authors declare no conflicts of interest.

## Supporting Information

Additional supporting information can be found online in the Supporting Information section.

## Supporting information


**Supporting Information 1** Figure S1: ceRNA regulatory network analysis (A) Venn diagram showing the intersection of miRNAs predicted by DIANA‐microT (*n* = 106) and TargetScan (*n* = 110) databases, yielding 29 common miRNAs. (B) A Sankey diagram illustrating the regulatory network of lncRNA‐miRNA‐mRNA for CD274, HBEGF, and IRS1.

**Supporting Information 2** Figure S2: Drug–gene interaction network The network shows 60 predicted drugs (circular nodes) and their interactions with five candidate key genes (diamond nodes: IRS1, HBEGF, MYC, CD274, and HMOX1). Node color represents the interaction score (darker red indicates higher score).

**Supporting Information 3** Figure S3: GSEA results for IRS1, HBEGF, CD274, and HMOX1 dot plots showing the top positively correlated (red) and negatively correlated (blue) KEGG pathways for each candidate key gene. The size of the dot indicates the number of genes, whereas the color signifies the adjusted *p* value.

**Supporting Information 4** Table S1: Predicted drugs targeting the five candidate key genes.

## Data Availability

The current study included data generated and analyzed by the authors.
